# Extracorporeal shock wave lithotripsy treatment of pancreatic stones complicated with advanced stage autoimmune pancreatitis

**DOI:** 10.1186/s12876-015-0255-9

**Published:** 2015-03-10

**Authors:** Masahiro Maruyama, Takayuki Watanabe, Keita Kanai, Takaya Oguchi, Jumpei Asano, Tetsuya Ito, Takashi Muraki, Hideaki Hamano, Norikazu Arakura, Takeshi Uehara, Shigeyuki Kawa

**Affiliations:** 1 Department of Gastroenterology, Shinshu University School of Medicine, 3-1-1 Asahi, Matsumoto, 390-8621 Japan; 2 Endoscopic Examination Center, Shinshu University School of Medicine, 3-1-1 Asahi, Matsumoto, 390-8621 Japan; 3 Department of Laboratory Medicine, Shinshu University School of Medicine, 3-1-1 Asahi, Matsumoto, 390-8621 Japan; 4 Center for Health, Safety, and Environmental Management, Shinshu University, 3-1-1 Asahi, Matsumoto, 390-8621 Japan

**Keywords:** Autoimmune pancreatitis, Chronic pancreatitis, Pancreatic stone, Pancreatic calculi, Extracorporeal shock wave lithotripsy, Endoscopic retrograde cholangiopancreatography

## Abstract

**Background:**

Although most patients with autoimmune pancreatitis (AIP) respond favorably to prednisolone therapy, some individuals who later suffer from pancreatic calculi may require additional extracorporeal shock wave lithotripsy (ESWL) treatment. This study compares the efficacy of ESWL for calculi in AIP with that in ordinary chronic pancreatitis (CP) and proposes a new treatment approach for pancreatic duct stones occurring in AIP.

**Methods:**

We examined the clinical records of 8 patients with chronic stage AIP and 92 patients with ordinary CP who received ESWL for pancreatic calculi.

**Results:**

The AIP group was significantly older than the CP group (69.0 vs. 56.5 years, *P* = 0.018). With regard to the indications for ESWL, chronic pain was significantly less frequent in the chronic stage AIP group (0% vs. 45.7%, *P* = 0.001), whereas preservation of pancreatic function was significantly more frequent (75% vs. 19.6%, *P* = 0.001). Compared with the CP group, the AIP group tended to exhibit pancreatic duct stenosis proximal to pancreatic calculi and had a lower rate of complete extraction of stones from the main pancreatic duct. Histopathological analysis of a patient with chronic stage AIP revealed widely distributed nodular pancreatitis, which was characteristic of ordinary CP, along with isolated areas of lymphoplasmacytic sclerosing pancreatitis.

**Conclusions:**

Different approaches are needed for the treatment of pancreatic calculi in chronic stage AIP and ordinary CP. Specifically, it appears that intensive ESWL therapy can be avoided or delayed in AIP if the patient displays: (1) advanced age, (2) little or no chronic pain or pancreatitis, and (3) pancreatic duct stenosis proximal to pancreatic stones. In such cases, the benefit of ESWL treatment may be outweighed by the risks involved in this procedure.

## Background

Autoimmune pancreatitis (AIP) has been recognized as a distinct type of pancreatitis that is possibly caused by autoimmune mechanisms [1-3]. AIP is characterized by pancreatic enlargement and irregular narrowing of the main pancreatic duct (MPD), both of which mimic the imaging features of pancreatic cancer [4-8]. Other prominent manifestations in AIP include high serum IgG4 concentration and IgG4-positive plasma cell infiltration in the affected pancreatic tissue, which are useful for serological and pathological diagnosis, respectively [9-11]. Patients with AIP respond favorably to prednisolone (PSL) therapy from the clinical, serological, imaging, and pathological perspectives [12-16].

In 1995, Yoshida et al. first proposed a concept of AIP that did not include pancreatic calcification [4]. As AIP patients responded favorably to corticosteroid therapy, the disease was believed to be a non-progressive condition that did not lead to pancreatic stone formation, which was a characteristic feature of advanced stage chronic pancreatitis (CP). Later, it was discovered that some patients with AIP experienced pancreatic calculi formation, pancreatic atrophy, and/or irregular dilatation of the MPD over a long-term course [14,17-26]. Such imaging findings corresponded to those of CP, suggesting that AIP could progress to a chronic state. We earlier reported that pancreatic calcification was closely associated with relapse and that AIP could develop into confirmed CP after multiple recurrences [17]. Hart et al. later conducted an international survey of AIP and found that 46 of 659 patients (7%) experienced pancreatic calcification, which supported the hypothesis that a chronic condition was significantly more frequent in relapse than in non-relapse individuals [14]. We also discovered that a primary risk factor for pancreatic stone formation in AIP was narrowing of both Wirsung’s and Santorini’s ducts in the pancreatic head region at the time of diagnosis [27]. Furthermore, we identified that the major risk factors for AIP developing into CP that satisfied the Revised Japanese Clinical Diagnostic Criteria (JCDC) for Ordinary Chronic Pancreatitis [28] were pancreatic head swelling and MPD non-narrowing in the pancreatic body [29]. Collectively, these findings suggested that AIP could progress to confirmed CP with severe pancreatic stone formation over a long-term period, most presumably due to disease recurrence and pancreatic juice stasis from remnant pancreatic duct stenosis [30].

Extracorporeal shock wave lithotripsy (ESWL) is generally considered to be effective for the treatment of pancreatic duct stones in ordinary CP [31-36]. However, there are few reports on the treatment of pancreatic duct calculi in AIP, and an effective therapeutic course has not been fully established owing to the different pathophysiologies of AIP and ordinary CP. Many patients with AIP experience pancreatic head swelling during the acute stage that leads to remnant narrowing of the MPD in the region [27,29]. This pathological condition creates problems in the drainage of crushed pancreatic stones, even after ESWL, and reduces the therapeutic effectiveness of pancreatic stone removal in AIP. Accordingly, an alternative perspective on for the treatment of pancreatic duct stones in AIP is needed. This study evaluated whether the efficacy of ESWL treatment for pancreatic duct stones in AIP was comparable with that in ordinary CP and proposed a new therapeutic approach for chronic stage AIP patients.

## Methods

### Study subjects

This retrospective study examined the ESWL records of 8 patients with chronic stage AIP and 92 patients with ordinary CP that were obtained between March 1996 and August 2012 at Shinshu University Hospital. During the study period, 73 AIP patients were registered at our hospital, which included 56 men and 17 women (median age: 66 years, range: 38–84 years). Of them, 63 patients (86%) received steroid therapy and 15 patients (20%) experienced calculi formation that met the Japanese diagnostic criteria for ordinary CP. The indications for ESWL were the following: (1) obstructing stone in the MPD whose volume was deemed too large for endoscopic therapy or for which therapeutic endoscopy had already been unsuccessful, (2) chronic pain or repeated pancreatitis attacks, and (3) preservation of pancreatic function by pancreatic juice release. Pancreatic function in the context of this study was defined as the maintenance of a normal pancreatic condition, including the absence of advanced diabetes mellitus and severe indigestion due to pancreatic dysfunction. Patients with malignancies were excluded following extensive examination using endoscopic ultrasound-guided fine needle aspiration (EUS-FNA) for any suspected cases. ESWL was performed by two or more pancreatology experts using a Piezolith 2500 lithotriptor (Piezoelectric effect technique; Richard Wolf GmbH, Knittlingen, Germany) before 2004 and a LITHOSTAR Multiline (Electromagnetic generation technique; Siemens GmbH, Munich, Germany) afterwards. ESWL sessions consisted of 3000 shocks over 60 minutes and were performed twice per week. Technical success of ESWL was defined as: (1) adequate stone fragmentation allowing extraction by therapeutic endoscopy or (2) the absence of targeted stones in follow-up radiographs.

For the treatment of chronic calcified pancreatitis, imaging tests, including ultrasound, CT, MRI, and/or endoscopic ultrasound, were first performed to confirm stone and duct morphology. Next, diagnostic endoscopic retrograde cholangiopancreatography (ERCP) was conducted to identify the stones obstructing the MPD. Therapeutic endoscopy was initially attempted for all cases. Endoscopic pancreatic sphincterotomy (EPST) was performed prior to ESWL whenever possible. Treated stones were primarily identified by the above imaging modalities. However, when other radiolucent stones were detected by ERP that met the indications for ESWL, a nasopancreatic tube was inserted to visualize the stones as negative images using contrast material to aid in ESWL therapy. Therapeutic ERCP was done for all patients within 4 days after ESWL. The clearance of residual fragmented stones in the MPD was performed by endoscopic pancreatolithotripsy mainly via a conventional basket. Endoscopic pancreatic stenting (EPS) was carried out if dominant strictures were present or when MPD clearance was deemed as inadequate.

We classified cases of CP as either chronic stage AIP or ordinary CP according to disease etiology. AIP diagnosis was based on the International Consensus Diagnostic Criteria (ICDC) for AIP [37], and all patients were diagnosed as having type 1 AIP. The diagnosis of ordinary CP was made according to the Revised JCDC for Ordinary Chronic Pancreatitis [28], in which severe pancreatic stone formation and marked calcification were the main diagnostic items. To clarify the differences in efficacy of ESWL pancreatic stone treatment between chronic stage AIP and ordinary CP, we compared clinical features, treatment details, and outcomes.

### Statistical analysis

Fisher’s exact and Pearson’s chi-square tests were adopted to test for differences between subgroups of patients. The Mann–Whitney U test was employed to compare continuous data. Multivariate analyses were performed using a logistic regression model. All tests were done using StatFlex ver. 6 software for Windows (Artec, Osaka, Japan). *P* values of less than 0.05 were considered to be statistically significant.

### Ethics

This study was approved by the ethics committee of Shinshu University.

## Results

### Clinical features

The clinical findings of the 8 patients (7 men and 1 woman) with chronic stage AIP having undergone ESWL for pancreatic stones are shown in Table 1. The median age and follow-up were 69 years (range: 59–73 years) and 68 months (range: 36–180 months), respectively. Seven patients (87.5%) had elevated serum IgG4 concentration, 5 patients (62.5%) were being treated with prednisolone (PSL), and 4 patients (50%) had experienced a relapse during the study period. The 92 patients with ordinary CP, which included 70 patients with alcoholic CP and 22 with idiopathic CP, presented at a median age of 56.5 years (range: 20–85 years) and consisted of 77 men and 15 women.

**Table 1:** **Characteristics of 8 patients with chronic stage autoimmune pancreatitis**

Case	Age	Gender	Serum IgG4	Steroid treatment	Relapse
	(years)	(male/female)	(mg)	(+/−)	(+/−)
1	70	M	725	+	+
2	64	M	965	+	+
3	66	M	730	+	+
4	73	M	185	-	-
5	68	F	1110	+	-
6	59	M	352	-	-
7	71	M	36	-	-
8	73	M	229	+	+

Univariate analysis for comparisons between chronic stage AIP and ordinary CP groups revealed that the AIP group was significantly older (69.0 vs. 56.5 years, *P* = 0.018). A tendency for male preponderance was seen in both groups. Regarding indications for ESWL therapy, chronic pain was significantly less frequent in the chronic stage AIP group (0% vs. 45.7%, *P* = 0.001), while preservation of pancreatic function was significantly more frequent (75% vs. 19.6%, *P* = 0.001) (Table 2).

**Table 2:** **Comparison of clinical features, treatment details, and outcomes between patients with autoimmune pancreatitis and chronic pancreatitis**

	AIP (n = 8)	CP (n = 92)	*P*value
**Clinical features**	**Median (range)**		
Age (years)	69.0 (59–73)	56.5 (20–85)	0.018^*^
Gender (male/female)	7/1	77/15	1.000
Therapeutic purpose for ESWL			
Chronic pain (+/−)	0/8	42/50	0.019^*^
Pancreatic attack (+/−)	1/7	32/60	0.265
Preservation of pancreatic function (+/−)	7/1	18/74	<0.001^*^
**Treatment details**			
Location of treated pancreatic stones			
Pancreatic head	6/2	83/9	0.213
Pancreatic body	3/5	15/77	0.153
Pancreatic tail	0/8	1/91	1.000
Pancreatic duct stenosis proximal to stones (+/−)	4/4	22/70	0.107
Endoscopic treatment	6/2	66/26	1.000
Endoscopic pancreatic sphincterotomy (+/−)	3/5	39/53	1.000
Endoscopic pancreatolithotripsy (+/−)	3/5	45/47	0.716
Endoscopic pancreatic stenting (+/−)	3/5	32/60	1.000
**Outcomes**			
Extraction of pancreatic stones in MPD (+/−)	5/3	71/21	0.394
Shift to surgical treatment (+/−)	1/7	3/89	0.284
Complications associated with ESWL (+/−)	0/8	8/84	1.000
Relapse of pancreatic stones in MPD (+/−)	1/7	22/70	0.678

AIP: autoimmune pancreatitis; CP; chronic pancreatitis; ESWL: extracorporeal shock wave lithotripsy; MPD: main pancreatic duct.

^*^P < 0.05.

### Treatment details

**Figure 1:**
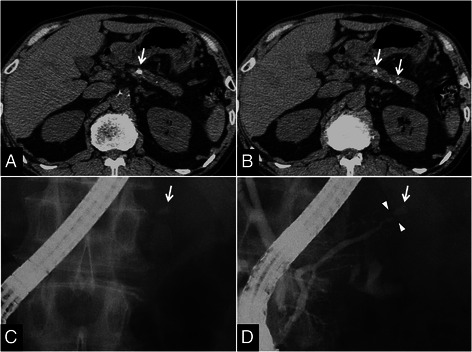
**CT and ERCP findings in a 66-year-old man whose pancreatic stone was treated with ESWL to preserve pancreatic function. (A)**, **(B)** CT before ESWL showing the pancreatic stone and pancreatic atrophy (arrows). **(C)**, **(D)** ERCP before ESWL identifying the obstructing X-ray-positive stone in the MPD (arrows) and pancreatic duct stenosis proximal to the pancreatic calculus (arrowheads). Pre-pancreatograpy **(C)** and post-pancreatography **(D)** images.

Stones located in the pancreatic head region were more frequently subjected to ESWL treatment in both chronic stage AIP and ordinary CP groups. However, pancreatic duct stenosis proximal to stones tended to be more frequent in the chronic stage AIP group (50% vs. 23.9%, *P* = 0.107) (Table 2) (Figure 1).

Concerning treatment regimen, there was no significant difference in the rate of combination ESWL and endoscopic treatment between the groups. We also found no appreciable differences in the rates of ESWL combined with individual endoscopic therapies, such as EPST, endoscopic pancreatolithotripsy, or EPS, between the groups (Table 2).

### Treatment outcomes

The complete extraction ratio of pancreatic stones from the MPD tended to be lower in the chronic stage AIP group than in the ordinary CP group (62.5% vs. 77.2%, *P* = 0.394) (Table 2). As a Piezoelectric Lithotripter was used for 39 cases before 2004 and a comparable Electromagnetic Lithotripter was adopted for 61 cases afterwards, there were no remarkable differences in the success rates of fragmentation or complete extraction ratio.

**Figure 2:**
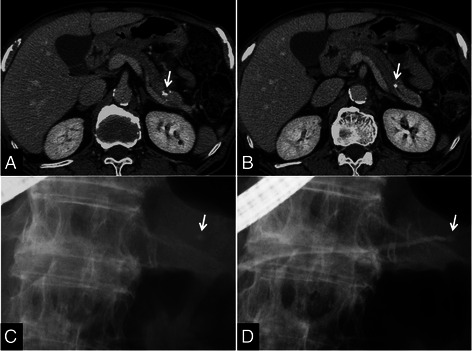
**CT and ERCP findings in a 71-year-old man who experienced a pancreatic attack relapse and was treated with ESWL. (A)**, **(B)** Contrast-enhanced CT before ESWL showing the pancreatic stone (arrows). **(C)**, **(D)** ERCP before ESWL identifying the obstructing X-ray-positive stone in the MPD (arrows). Pre-pancreatograpy **(C)** and post-pancreatography **(D)** images.

A shift from medical to surgical treatment occurred in 1 patient (12.5%) with chronic stage AIP and 3 (3.3%) with ordinary CP. This difference was not significant (12.5% vs. 3.3%, *P* = 0.284) (Table 2). Surgical treatment consisted of distal pancreatectomy in the AIP patient (Figure 2) and distal pancreatectomy and pancreaticojejunostomy in 1 and 2 CP patients, respectively.

Eight patients (8.7%) with ordinary CP experienced complications related to ESWL, as compared with none with chronic stage AIP (0%). These rates were comparable (0% vs. 8.7%, *P* = 0.623) (Table 2). ESWL complications were defined as events that occurred within several days after the procedure and were classified as either major, which needed further intervention, or minor, which were relieved by conservative treatment. We witnessed 4 of each complication type. Among the 4 cases of major complications, 2 patients experienced lower bile duct stricture that required endoscopic bile duct stenting and choledochojejunostomy, respectively. The remaining 2 patients displayed an infected pancreatic cyst needing endoscopic transpapillary drainage. All 4 minor complications were of mild acute pancreatitis that improved during observation.

The recurrence of pancreatic stones in the MPD was seen in 1 patient (12.5%) in the chronic stage AIP group at 56 months postoperatively and 22 patients (23.9%) with ordinary CP. This difference was not significant (12.5% vs. 23.9%, *P* = 0.678) (Table 2).

### Pathological analysis

We reviewed the postoperative histopathology of a patient with chronic stage AIP who displayed a stone in the pancreatic tail, suffered from repeated pancreatic attacks, experienced several recurrences of pancreatic stones following extraction by combination ESWL and endoscopic therapy, and ultimately underwent distal pancreatectomy to alleviate his symptoms (Figure 2).

**Figure 3:**
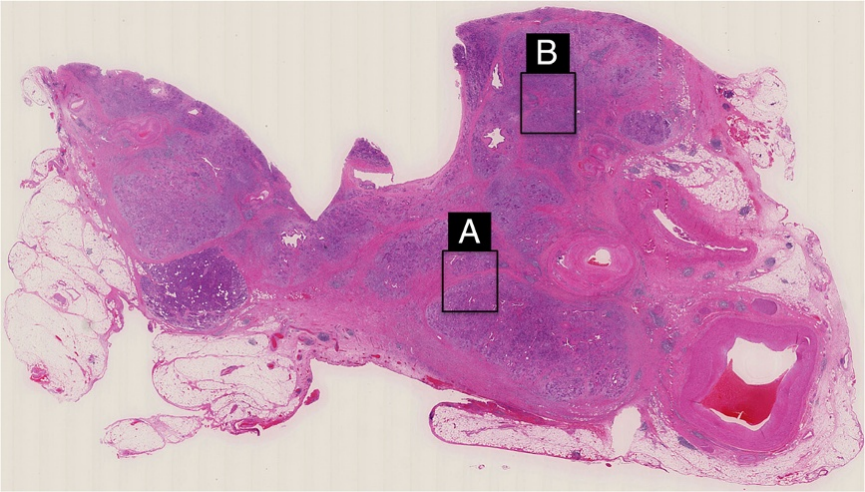
**Loupe image of chronic stage AIP (hematoxylin and eosin staining).** Pancreatic parenchyma exhibited abundant interlobular fibrosis that resembled multiple tuberosities with sclerotic variation **(A)** along with several areas of lymphoplasmacytic sclerosing pancreatitis (LPSP), in which interlobular fibrosis was unclear **(B)**.

**Figure 4:**
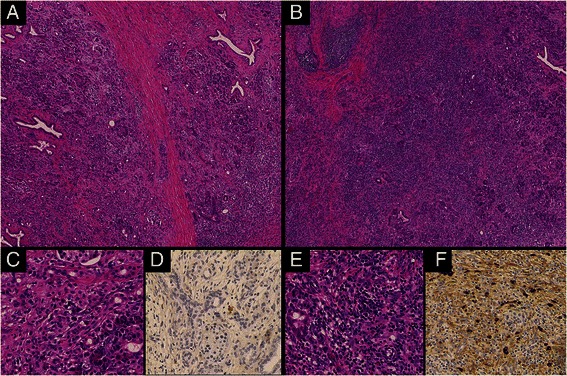
**Histological image of chronic stage AIP. (A)**, **(C)**, **(D)** Characteristics of ordinary CP. Mild inflammatory cell invasion, acinar atrophy, and slight IgG4-positive plasma cell infiltration are present in intralobular areas. Thick fibrosis is observed in interlobular areas. **(B)**, **(E)**, **(F)** Characteristics of AIP. Dense plasma cell and lymphocyte invasion, storiform fibrosis, obliterative phlebitis, and abundant IgG4-positive plasma cell infiltration exist in intralobular areas. Opaque fibrosis is present in interlobular areas. Hematoxylin and eosin staining, low-power field (×40) **(A, B)**, hematoxylin and eosin staining, high-power field (×400) **(C, E)**, and IgG4 immunostaining, high-power field (×400) **(D, F)** images.

In loupe images, the patient’s pancreatic parenchyma exhibited abundant interlobular fibrosis that resembled multiple tuberosities with sclerotic variation in addition to several areas with lymphoplasmacytic sclerosing pancreatitis (LPSP) in which interlobular fibrosis was unclear (Figure 3). In the former regions, several characteristic findings of ordinary CP, such as mild inflammatory cell infiltration, acinar atrophy, and scarce IgG4-positive plasma cell infiltration, were present in intralobular areas. Thick fibrosis was seen in interlobular areas. In contrast, the latter regions displayed characteristic AIP findings, including dense plasma cell and lymphocyte invasion, storiform fibrosis, obliterative phlebitis, and abundant IgG4-positive plasma cell infiltration in intralobular areas and opaque fibrosis in interlobular areas (Figure 4).

## Discussion

### Is pancreatic calculus treatment of AIP different from that of ordinary CP?

The present study uncovered the following observations regarding ESWL treatment of pancreatic calculi in patients with chronic stage AIP or ordinary CP: (1) the AIP group was significantly older than the CP group and displayed fewer clinical symptoms requiring ESWL therapy, (2) the AIP group showed frequent pancreatic duct stenosis proximal to pancreatic calculi, (3) the rate of complete stone extraction from the MPD was slightly lower in the AIP group, and (4) there were no significant differences in the rates of subsequent surgical treatment, adverse effects, or pancreatic stone recurrence after ESWL between the groups.

In this investigation, we enrolled 100 patients with chronic calcified pancreatitis who had undergone ESWL. The indications for ESWL were: (1) obstructing stone in the MPD whose volume was deemed too large for endoscopic therapy and (2) chronic pain or repeated pancreatitis attacks, as well as (3) preservation of pancreatic function by pancreatic juice release. All patients provided informed consent for this treatment.

Of the 73 patients with AIP who were registered at our hospital, 15 (20%) later experienced calculi formation that met the Japanese diagnostic criteria for ordinary CP. Among them, several patients who fulfilled the indications for ESWL did not consent to treatment, which was a limitation of this study. As we aimed to evaluate pancreatic stone treatment, we selected only the 8 patients with chronic stage AIP who had met the requirements for ESWL and had provided consent after being explained all possible complications, such as acute pancreatitis, and the risk of non-preservation of pancreatic function. Larger prospective comparisons of chronic stage AIP and ordinary CP are required.

Many patients with calcified ordinary CP complain of epigastralgia and back pain due to increased pancreatic duct pressure caused by intraductal pancreatic stones [38]. However, this study revealed that only 12% of AIP patients who were treated with ESWL displayed these symptoms. We earlier identified severe inflammation of the pancreatic head and non-narrowing of the pancreatic duct in the body region, both of which indicated that severe pancreatic juice stagnation had induced pancreatic calcification, to be risk factors for extensive pancreatic stone formation in AIP [27,29]. Accordingly, we had expected that AIP complicated with numerous pancreatic calculi would be accompanied by epigastralgia similarly to ordinary CP, but the present study showed that most AIP patients were asymptomatic. Although AIP and ordinary CP both displayed the imaging findings of severe pancreatic calculi and pancreatic atrophy that were characteristic of a chronic stage of pancreatitis, there was a discrepancy in the occurrence of abdominal pain between the two conditions. The reason for this difference may be pathophysiological differences that require further study.

In our cohort, ESWL was performed on AIP patients mainly to preserve pancreas function. Previous reports have examined the efficacy of pancreatic calculus treatment by endoscopy and ESWL on pancreatic exocrine and endocrine function in ordinary CP. In terms of exocrine function, a BT-PABA test showed improvement in 60-77% of cases [32,33], although several studies found no significant differences before and after therapy. Concerning endocrine function, few reports have been able to demonstrate a clear improvement in glucose tolerance or insulin secretion capacity following treatment [34,39]. In patients with chronic calcified pancreatitis who receive treatment for the purpose of function preservation, it will be of merit to evaluate whether pancreatic condition is affected by relevant therapy. However, as this study focused primarily on pancreatic stone treatment approaches, detailed pancreatic function readings were not obtained before and after intervention. The BT-PABA test is the standard pancreatic exocrine function examination in Japan, but it is affected by various factors, such as liver and renal dysfunction, and is somewhat complex for patients to understand. It will be important to perform precise assessment of exocrine and endocrine dysfunction in chronic stage AIP over a long-term period that includes the presence or absence of pancreatic stone treatment. We have also been considering new alternative approaches to the BT-PABA test.

We previously proposed that AIP could exhibit severe pancreatic stone formation over a long-term period due to disease recurrence [17,20] and pancreatic juice stasis preceded by pancreatic head swelling, narrowing of both Wirsung’s and Santorini’s ducts in the affected region, and MPD non-narrowing in the pancreatic body [27,29,30]. Another risk factor for pancreatic calculus formation in AIP is excessive alcohol intake of pure ethanol of >50 g/day [40]. There was 1 alcoholic subject among the 8 AIP patients who received calculus treatment with ESWL. In this patient, both pancreatic juice stagnation due to AIP-specific inflammation [27,29] and pancreatic juice denaturation from alcohol abuse might have been associated with the calculi. Further examination is required on alcohol consumption and the clinical background of pancreatic stone formation in AIP.

When assessing the suitability of ESWL treatment, it is important to identify patients having cancer of the pancreas. Subjects with pancreatic cancer were excluded from this investigation after extensive examination, although it should be noted that pancreatic stones make it challenging to detect pancreatic tumors. All ESWL patients were free from pancreatic cancer during the entire study period.

### What are effective approaches for the treatment of AIP with ESWL?

The present study uncovered a tendency for increased pancreatic duct stenosis proximal to pancreatic stones in AIP that was unlike the widely distributed duct stenosis encountered in ordinary CP. In such AIP patients, the stones fragmented by ESWL may sometimes have difficulty passing through the narrowed duct in the head region, which might diminish the efficacy of endoscopic treatment. Pancreatic duct dilation is a useful technique to remove crushed calculi pieces following ESWL in ordinary CP with pancreatic duct stenosis. Here, duct stenosis proximal to pancreatic calculi was present in 4 of 8 AIP patients (50%). Endoscopic pancreatic duct dilation was performed on 1 patient, which resulted in complete stone extraction. Thus, similarly to ordinary CP, combination therapy of endoscopic pancreatic duct dilation and ESWL in AIP may constitute an effective procedure to remove pancreatic stones in the presence of proximal duct stenosis.

We observed that pancreatic calculus treatment in AIP was significantly more common in elderly people who exhibited fewer symptoms in the present study. Accordingly, intensive ESWL and endoscopic treatment may be avoided or postponed in patients with the factors of: (1) advanced age, (2) mild or no chronic pain or pancreatitis, and (3) pancreatic duct stenosis proximal to pancreatic calculi. For such cases, we suggest conservative follow-up that includes periodic blood tests and imaging studies. Regular evaluation of exocrine and endocrine function during long-term follow-up will also help assess the need and timing of ESWL and endoscopic treatment in chronic AIP patients with pancreatic stones.

In ordinary CP, the most important factor in preventing calculus recurrence is avoidance of alcohol. However, treatment for pancreatic duct stenosis is thought to be another important step [41]. In AIP, MPD stenosis may affect not only the efficacy of pancreatic stone treatment, but also pancreatic stone recurrence afterwards. Furthermore, previous studies have reported that smoking status (not smoking or cessation) was related to the efficacy of ESWL and pain relief after ESWL for CP [36,42]. Although this investigation did not evaluate smoking habits, further examination is needed in comparisons between ordinary CP and chronic stage AIP. Careful follow-up to evaluate calculus recurrence and exacerbation is also required for AIP with pancreatic stones, regardless of any ESWL or endoscopic treatment.

### Is the histopathology of chronic stage AIP different from that of ordinary CP?

Although ordinary CP and chronic stage AIP exhibit similar imaging findings, including pancreatic calculus formation and pancreatic atrophy, their clinical manifestations, such as chronic pain and pancreatitis attacks, appear to be different. From the viewpoint of long-term pancreatic exocrine and endocrine function, it will be of interest to clarify whether the histopathology of ordinary CP is in fact different from that of chronic stage AIP with pancreatic stones. Since there have been few reports describing this relationship, we examined the pancreatic histopathology of an AIP patient who experienced pancreatic calculus relapse after surgical treatment and compared it with that of typical ordinary CP. We observed that the nodular pancreatitis characteristic of ordinary CP was widespread in tissue samples, while LPSP, which was typical of AIP, was found in restricted areas only. From these findings, we considered the following possibilities as mechanisms of chronic stage AIP histopathology: (1) based on our previous reports that AIP could progress to CP with severe calcification over a long-term period [27,29], LPSP may have shifted to a histopathology similar to that of ordinary CP, (2) due to the patient’s history of alcoholism, LPSP may have been complicated with a histopathology of typical alcoholic pancreatitis, and (3) based on the findings of Fukui et al. that obstructive pancreatitis complicated with pancreatic cancer also revealed abundant IgG4-bearing plasma cell infiltration [43], LPSP with marked IgG4-bearing plasma cell infiltration may have coexisted with obstructive pancreatitis. Although we followed the clinical outcome of a single patient, our findings suggested that AIP could shift to a clinical condition similar to that of ordinary CP not only in imaging findings, but also in pancreatic histopathology, over a long-term course. Further analysis of the pancreatic histopathology of AIP with pancreatic atrophy and calculi is needed to clarify the clinical conditions of advanced stage AIP.

In the present study, there are several limitations and future perspectives. Specifically, our investigation included a limited number of patients and was retrospective in nature. It also employed the revised JCDC for CP, which has a strong emphasis on imaging findings, and the subjects enrolled all had type 1 AIP. More detailed analyses of physical findings and exocrine and endocrine dysfunction are needed as well.

## Conclusions

The present study presented the clinical features and outcomes of patients with AIP who underwent ESWL treatment for severe pancreatic calculi. We postulate from our results using a limited number of patients that the approach for pancreatic stone treatment in AIP may be different from that in CP, whereby intensive ESWL treatment may be avoided or delayed if patients show: (1) advanced age, (2) little or no chronic pain or pancreatitis, and (3) pancreatic duct stenosis proximal to pancreatic calculi. In such cases, the benefit of ESWL treatment may be outweighed by the risks involved in this procedure.

## Abbreviations

AIP: Autoimmune pancreatitis
CP: Chronic pancreatitis
MPD: Main pancreatic duct
PSL: Prednisolone
ESWL: Extracorporeal shock wave lithotripsy
ERCP: Endoscopic retrograde cholangiopancreatography
EPST: Endoscopic pancreatic sphincterotomy
EPS: Endoscopic pancreatic stenting
ICDC: International consensus diagnostic criteria
JCDC: Japanese clinical diagnostic criteria
LPSP: Lymphoplasmacytic sclerosing pancreatitis
